# The development and validation of a microneutralization assay for the detection and quantification of anti-yellow fever virus antibodies in human serum

**DOI:** 10.1128/spectrum.03348-24

**Published:** 2025-03-04

**Authors:** Katherine Fries, Ping Luo, Rebekah Baldwin, Raechel Goldberg, Ivan Ordonez, Lingyi Zheng, James Huleatt, Louis Devlin

**Affiliations:** 1 Global Clinical Immunology, Sanofi, Swiftwater, Pennsylvania, USA; 2 Translational and Early Development Biostatistics, Sanofi, Orlando, Florida, USA; 3 Translational and Early Development Biostatistics, Sanofi, Swiftwater, Pennsylvania, USA; Naturwissenschaftliches und Medizinisches Institut an der Universitat Tubingen, Reutlingen, Germany

**Keywords:** assay development, assay validation, microneutralization assay, neutralizing antibodies, orthoflavivirus, yellow fever virus

## Abstract

The plaque reduction neutralization test (PRNT) has been used widely for the detection and quantitation of yellow fever (YF) virus-neutralizing antibodies in human serum; however, it is labor-intensive and challenging to adapt to high-throughput clinical testing needed for vaccine licensure. Here, we describe the development and validation of a new Vero cell-based YF microneutralization (MN) assay, with immunostaining readout, for the detection and quantification of YF virus-neutralizing antibodies in human serum. Comparison of neutralizing antibody titers measured with the YF MN assay versus the historical YF PRNT, based on a 50% reduction in plaque counts (PRNT_50_), demonstrated 100% serostatus agreement at a titer of 10 (1/dil) in participants with a history of YF vaccination. For validation, intra-assay precision (repeatability), intermediate precision, dilutional accuracy, linearity, specificity, upper limit of quantitation (ULOQ), and lower limit of quantitation (LLOQ) were assessed. The YF MN assay demonstrated suitable intra-assay precision (repeatability) and intermediate precision of 36% and 54%, respectively, with an ULOQ of 10,240. At the lower end of detection, repeatability and intermediate precision were 38% and 41%, respectively, with a LLOQ of 10 (1/dil). Suitable dilutional accuracy, linearity, and specificity across orthoflaviviruses (dengue virus, Japanese encephalitis virus, and Zika virus) and serum matrices (hemolytic, lipemic, and icteric) were also demonstrated. Overall, these promising results led the Center for Biologics Evaluation and Research to confirm the suitability of the validated YF MN assay for the detection and quantification of YF virus-neutralizing antibodies.

**IMPORTANCE:**

With increased globalization and shifting climate patterns, yellow fever (YF) is re-emerging as a global threat. At present, vaccination remains the most effective prevention strategy. This study describes the development and validation of a new YF microneutralization (MN) assay for the detection and quantification of YF virus-neutralizing antibodies in human serum that offers increased throughput compared with the current standard assay. Overall, the YF MN assay demonstrated acceptable intra-assay precision (repeatability), intermediate precision, dilutional accuracy, linearity, and specificity and is suitable for the detection of YF virus-neutralizing antibodies. Further, the Center for Biologics Evaluation and Research (CBER) supports the use of the YF MN assay in the licensure of candidate YF vaccines.

## INTRODUCTION

Yellow fever (YF) virus is a mosquito-borne virus transmitted to humans primarily through the bite of infected *Aedes* and *Haemagogus* species of mosquitos (1). The viral etiology of YF disease is an arbovirus, single-stranded, positive ribonucleic acid member of the genus, Orthoflavivirus, recently renamed from Flavivirus (2). Owing to the presence of competent hosts, vectors, and the etiological agent, YF disease is endemic in tropical and subtropical areas of Africa, and Central and South America, and is driven by three unique transmission cycles (jungle, savannah, and urban) (3–5). Although most YF infections are asymptomatic, infection can also lead to the development of mild, self-limited febrile illness, which can, in some cases, progress to severe hemorrhagic fever, leading to death (4–6). In 2018 alone, there were 109,000 (95% credible interval [CrI] 67,000–173,000) severe infections and 51,000 (95% CrI 31,000–82,000) deaths (7), with about 90% of the burden occurring in Africa (3, 4). To date, no drug has demonstrated specific benefits in the treatment of YF, and disease management remains supportive. Safe and effective live attenuated YF vaccines based on the YF sub-strain, 17D, have been available since the 1930s and have been central in disease control (8).

Although “traditional” YF vaccine manufacturing provides a reliable vaccine, the process is slow and labor-intensive and may be limited by the availability of pathogen-free embryonated hens’ eggs; this, in part, has led to insufficient YF vaccine supply globally due to increased demand through outbreak responses and continued routine and preventative campaign requirements (9, 10). By contrast, cell-based manufacturing provides a fast, sustainable platform, which also offers flexibility and improves production scalability and lead times (11). As part of the global effort to improve the supply of YF vaccine, Sanofi has developed a YF-17D vaccine produced in well-characterized Vero cells, cultured in media without products of animal origin. The new Vero cell-based candidate, YF vaccine (vYF), is currently entering phase III global clinical development (12, 13).

The serological diagnosis of YF virus infection includes the detection of either anti-YF virus-specific immunoglobulin (Ig) M antibodies or a fourfold or greater increase in anti-YF virus IgG antibody titers between acute and convalescent samples in the absence of recent vaccination. Several serological assays for the detection and quantification of YF virus are available for use in reference laboratories, including enzyme-linked immunosorbent assay, immunofluorescent antibody test, complement fixation, log_10_ neutralization index, and plaque reduction neutralization test (PRNT) (14–17). The YF virus can also be detected by culture, immunohistochemistry, or reverse transcription-polymerase chain reaction methods (14, 15, 18). However, the serological diagnosis of YF infection is complicated by cross-reactivity with other orthoflaviviruses, such as dengue and Zika (14, 15). The PRNT is generally considered the assay of choice as it offers better specificity but requires highly trained/experienced staff to undertake the assay and is unsuitable for high-throughput requirements (15, 19). As such, there is a need for robust high-throughput assays for both diagnostic surveillance and the assessment and licensure of candidate YF vaccines (14).

A Vero cell-based YF microneutralization (MN) assay with an immunostaining readout was developed for the detection and quantification of YF virus-neutralizing antibodies after immunization with YF vaccines. Here, we describe the development and validation of this new YF MN assay.

## RESULTS

### Qualification of YF MN internal quality controls

The establishment of valid titer ranges for the candidate internal quality controls (IQCs) is summarized in Table 1. More than 90% of the results for each candidate IQC sample were within the valid titer range, meeting the acceptance criteria. The p-value of the Shapiro–Wilk test, applied to the histograms and normality assessments, was >0.05 for each positive IQC1 and IQC2 candidate sample, indicating normally distributed results (Fig. S1).

**TABLE 1:** Qualification of YF MN assay IQCs^*a*^

IQC candidate		GMT (min–max)	LL (GMT - 2-fold)	UL (GMT + 2-fold)	Results within ±2-fold of GMT, % (n/N)	% GCV^*b*^
Anti-YF virus positive	IQC1 (*N* = 85)	669 (343–2,911)	334	1,338	97.6 (83/85)	43
	IQC2 (*N* = 86)	1,626 (919–4,038)	813	3,252	95.3 (82/86)	40
Anti-YF virus negative	IQC3 (*N* = 62)	<10 (<10–16)	N/A	<10	98.4^*c*^ (61/62)	N/A

^*a*^ %GCV, percentage of geometric coefficient of variation; GMT, geometric mean titer; IQC, internal quality control; LL, lower limit; MN, microneutralization; N, number of valid results used in the analysis; N/A, not applicable, SD, standard deviation; UL, upper limit; YF, yellow fever.

^*b*^ % GCV was calculated as (10^SD-1^)*100%, where SD was the SD of the log_10_-transformed results; however, current practice is to calculate % GCV as ($\sqrt{\exp(\text{ var })-1}$)*100%, where var is the sample variance of the natural log-transformed results; in such a case, the % GCV for IQC1 and IQC2 were 37% and 35%, respectively.

^*c*^ The precision of the negative IQC was assessed by the percentage of the results tested as negative (i.e., the titer of <10).

### Short-term stability of human serum samples

The short-term stability of YF virus neutralizing antibodies present in human serum samples after five freeze–thaw cycles or storage at 2°C to 8°C for up to 14 days was demonstrated. Overall, results were within twofold assay variability, and anti-YF virus-neutralizing antibodies in human serum samples were considered stable under the test conditions (Table 2).

**TABLE 2:** Short-term anti-YF virus antibody stability^*a*^

Sample	Treatment	Observed YF MN_50_	Expected titer	ABS log_2_ difference
IQC1 (positive)	No treatment	326	326	N/A
	Five freeze–thaw cycles	330		0.02
	14 days, 2°C to 8°C	413		0.34
IQC2 (positive)	No treatment	1,167	1,167	N/A
	Five freeze–thaw cycles	852		0.45
	14 days, 2°C to 8°C	878		0.41
IQC3 (negative)	No treatment	<10	<10	N/A
	Five freeze–thaw cycles	<10		N/A
	14 days, 2°C to 8°C	<10		N/A

^*a*^ ABS, absolute value; IQC, internal quality control; MN_50_, 50% microneutralization; N/A, not applicable; YF, yellow fever.

### Evaluation of serostatus agreement between YF MN assay and YF PRNT

A panel of 236 serum samples from healthy adults (Sanofi employees, *n* = 37; clinical serum samples from a phase II study, *n* = 199) were evaluated to compare the serostatus agreement at a titer of 10 (1/dil) (lower limit of quantitation [LLOQ]) between the YF MN assay, YF PRNT_50_ (50% reduction in viral plaques), and YF PRNT_80_ (80% reduction in viral plaques). These serum samples were from participants with (*n* = 142) or without (*n* = 94) a history of YF vaccination. Overall, for samples from those with a history of YF vaccination, serostatus agreement was 100% (142/142) between the YF MN assay and YF PRNT_50_, and 96.5% (137/142) between the YF MN assay and YF PRNT_80_. Notably, five samples determined as positive using both the YF MN assay and YF PRNT_50_ were classed as negative (<10, 1/dil) with YF PRNT_80_ (Table 3; Table S1). In contrast, for samples from those without a history of YF vaccination serostatus agreement was 34.0% (32/94) between the YF MN assay and PRNT_50_ and 100% (94/94) between the YF MN assay and YF PRNT_80_. Notably, 62 samples determined as seronegative (<10, 1/dil) using both the YF MN assay and YF PRNT_80_ were classed as positive with PRNT_50_ (Table 4; Table S2).

**TABLE 3:** Summary of serostatus agreement between the YF MN assay and YF PRNT in serum samples from participants with a history of YF vaccination^*a*^

		YF PRNT_50_		YF PRNT_80_	
		<10	≥10	<10	≥10
YF MN assay	<10	0	0	0	0
	≥10	0	142	5	137
Agreement (%)		100.0% (142/142)		96.5% (137/142)	

^*a*^ MN, microneutralization; PRNT_50_, 50% plaque reduction neutralization test; PRNT_80_, 80% plaque reduction neutralization test; YF, yellow fever.

**TABLE 4:** Summary of serostatus agreement between the YF MN assay and YF PRNT in serum samples from participants without a history of YF vaccination^*a*^

		YF PRNT_50_		YF PRNT_80_	
		<10	≥10	<10	≥10
YF MN assay	<10	32	62	94	0
	≥10	0	0	0	0
Agreement (%)		34.0% (32/94)		100% (94/94)	

^*a*^ MN, microneutralization; PRNT_50_, 50% plaque reduction neutralization test; PRNT_80_, 80% plaque reduction neutralization test; YF, yellow fever.

### Cross-reactivity

To evaluate the cross-reactivity of the YF MN assay with human serum samples containing anti-dengue virus (DENV) antibodies, a total of 38 samples from naturally DENV-infected individuals were tested using the YF MN assay and DENV PRNT. The anti-DENV antibody status of all 38 samples was determined to be positive with the DENV PRNT (Table 5). The anti-YF serostatus of the individuals was unknown; however, it was assumed to be negative as the samples were obtained from countries that are non-endemic for YF. Overall, 13% (5/38) of the anti-DENV-positive samples were determined to be low positive in the YF MN assay, with observed MN titers within two-fold of the LLOQ of 10 (1/dil; range: 11–17) (Table 5; Table S3). The observed YF MN assay and DENV PRNT results are shown in Table S3. Although a low percentage of cross-reactivity was observed with the YF MN assay, this cross-reactivity should not impact the outcome of epidemiologic or clinical studies focusing on YF. To evaluate the immune response against the YF candidate vaccine, vYF, anti-YF virus neutralizing antibody titers were measured in pre- and post-YF vaccination samples using the YF MN assay. Seroconversion was defined as a fourfold increase in neutralizing antibody titers as compared with the pre-vaccination value.

**TABLE 5:** YF MN assay cross-reactivity in DENV-positive samples^*a*^

Sample source	Clinical study sample	Commercial samples^*b*^	
Country of origin	India	San Salvador	Honduras
DENV infection history	Naturally infected	Naturally infected	Naturally infected
Anti-DENV status	Positive	Positive	Positive
Anti-YF virus status	Assumed negative	Assumed negative	Assumed negative
Total number of samples tested	35	2	1
Number of anti-YF neutralizing antibody negative samples	30	2	1
Number of samples identified as anti-YF neutralizing antibody positive	5	0	0

^*a*^ DENV, dengue virus; MN, microneutralization; YF, yellow fever.

^*b*^ Biomnis commercial sample from San Salvador or a SeraCare commercial sample from Honduras.

### YF MN assay validation

#### Intra-assay precision (repeatability) and intermediate precision

The YF MN assay demonstrated intra-assay precision (repeatability) of 36% and intermediate precision of 54%, both meeting the acceptance criterion for the percentage of geometric coefficient of variation (% GCV) of ≤60% (Table 6).

**TABLE 6:** Summary of the YF MN assay methods and validation acceptance criteria^*a*^

Parameter	Testing method	Acceptance criteria	Results
Intra-assay precision (repeatability)	50 human serum samples (including 10 incurred clinical samples) were tested in triplicate in three individual assay runs by three analysts (up to nine results per sample)	Repeatability: The overall % GCV had to be ≤60%	Repeatability: % GCV = 36%
Inter-assay precision (intermediate precision)		Intermediate precision: The overall % GCV had to be ≤60%	Intermediate precision: % GCV = 54%
			
ULOQ	12 high-titer samples were tested to define the ULOQ by testing at a serum starting dilution of 1:20 (2 × the method serum starting dilution) in triplicate in three individual assay runs by two analysts	The ULOQ was defined as the highest titer measured from a sample that met the acceptance criteria of precision (i.e., % GCV ≤60%) up to a titer of 10,240	ULOQ = 10,240 (highest titer from a sample with % GCV ≤60%) up to a value of 10,240
			
LLOQ	17 low-titer/negative samples (including six incurred clinical samples) with GMTs up to ~4 × the theoretical LLOQ were tested to confirm the LLOQ of 10 by testing at a starting dilution of 1:5 (half the method serum starting dilution) in triplicate in three individual assay runs by three analysts (up to nine results per sample)	The overall % GCV of the samples evaluated for LLOQ confirmation had to be ≤60%	LLOQ = 10 Repeatability % GCV = 38% Intermediate precision % GCV = 41%
			
Dilutional accuracy	Six anti-YF virus antibody-positive human serum samples (including three incurred clinical samples, undiluted, and prediluted 1:5, 1:10, 1:20, and 1:40 in either assay diluent or Ig-depleted human sera) were tested in triplicate in three individual assay runs (up to nine results per sample); linear regression was performed to assess linearity	The absolute difference of the log_2_-observed GMT and the log_2_-expected value had to be ≤1.00 for ≥80% of the samples	88% (21/24) of samples had an absolute log_2_ difference of ≤1.00
Linearity		≥80% of the samples had an R^2^ of ≥0.95, and the slope of the regression line was between 0.67 and 1.50	100% (6/6) of samples had a R^2^ > 0.95 and a slope between 0.67 and 1.50
			
Specificity spiking study	Six anti-YF virus antibody-positive human serum samples (including three incurred clinical samples) were spiked with anti-DENV, anti-JEV, or anti-ZIKV antibody-positive human serum samples, or anti-YF virus antibody-negative human serum (baseline control) and tested in triplicate in one assay run	The absolute difference between the log_2_ observed GMT and the log_2_ expected value had to be ≤1.00 for ≥80% of the samples	100% (6/6) of the spiked samples had an absolute log_2_ difference of ≤1.00
Specificity Matrix effect study	Six anti-YF virus antibody-positive human serum samples (including three incurred clinical samples) were spiked in different matrix samples (hemolytic, lipemic, icteric) or anti-YF virus-negative human serum (baseline control) and tested in triplicate in one assay run	The absolute difference between the log_2_ observed GMT and the log_2_ expected value had to be ≤1.00 for ≥80% of the samples	100% (6/6) of the spiked samples had an absolute log_2_ difference of ≤1.00

^*a*^ % GCV, percentage of geometric coefficient of variation; DENV, dengue virus; GMT, geometric mean titer; JEV, Japanese encephalitis virus; LLOQ, lower limit of quantification; MN, microneutralization; N/A, not applicable; ULOQ, upper limit of quantification; YF, yellow fever; and ZIKV, Zika virus.

#### Upper and lower limits of quantitation

**Fig 1:**
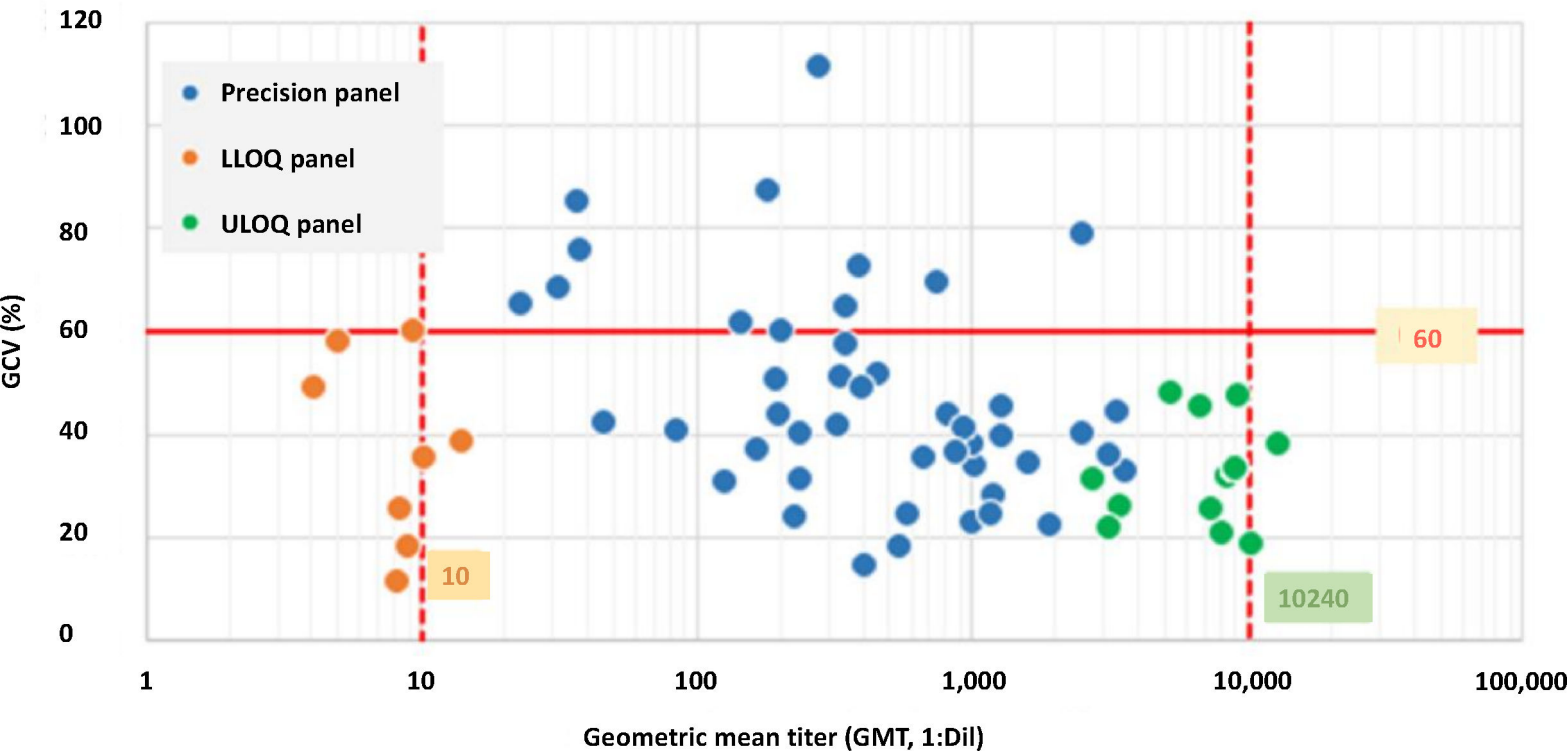
The YF MN assay combined precision profile graphically represents data from the precision panel (blue dots), generated by testing the precision samples at a 1:10 serum starting dilution, the LLOQ panel (orange dots), generated by testing the LLOQ samples at a 1:5 serum starting dilution, and from the ULOQ panel (green dots), generated by testing the ULOQ samples at a 1:20 serum starting dilution. The statistical analysis of the combined YF MN assay precision data demonstrated acceptable precision from the LLOQ titer of 10 to the ULOQ titer of 10,240. Abbreviations: Dil, dilution; GCV, geometric coefficient of variation; LLOQ, lower limit of quantitation; MN, microneutralization; ULOQ, upper limit of quantitation; and YF, yellow fever.

The YF MN assay maintained acceptable precision (GCV ≤60%) up to an upper limit titer of 10,240 (Table 6). For intra-assay precision (repeatability) and intermediate precision at the lower end of detection, statistical analysis demonstrated repeatability of 38% and intermediate precision of 41%, both meeting the acceptance criterion of % GCV ≤60%, with an LLOQ titer of 10 (1/dil) confirmed (Fig. 1).

#### Dilutional accuracy

The YF MN assay demonstrated suitable dilutional accuracy for the detection of YF virus-neutralizing antibodies as the absolute difference between the observed and expected log_2_ titers was ≤1.00 for 88% (21/24) of samples (Table 6). The three samples with an absolute difference of log_2_ > 1.00 were all samples prediluted to 1:40; therefore, the serum samples were only tested at the 1:10 serum starting dilution to prevent bias from using a higher serum starting dilution.

#### Linearity

**Fig 2:**
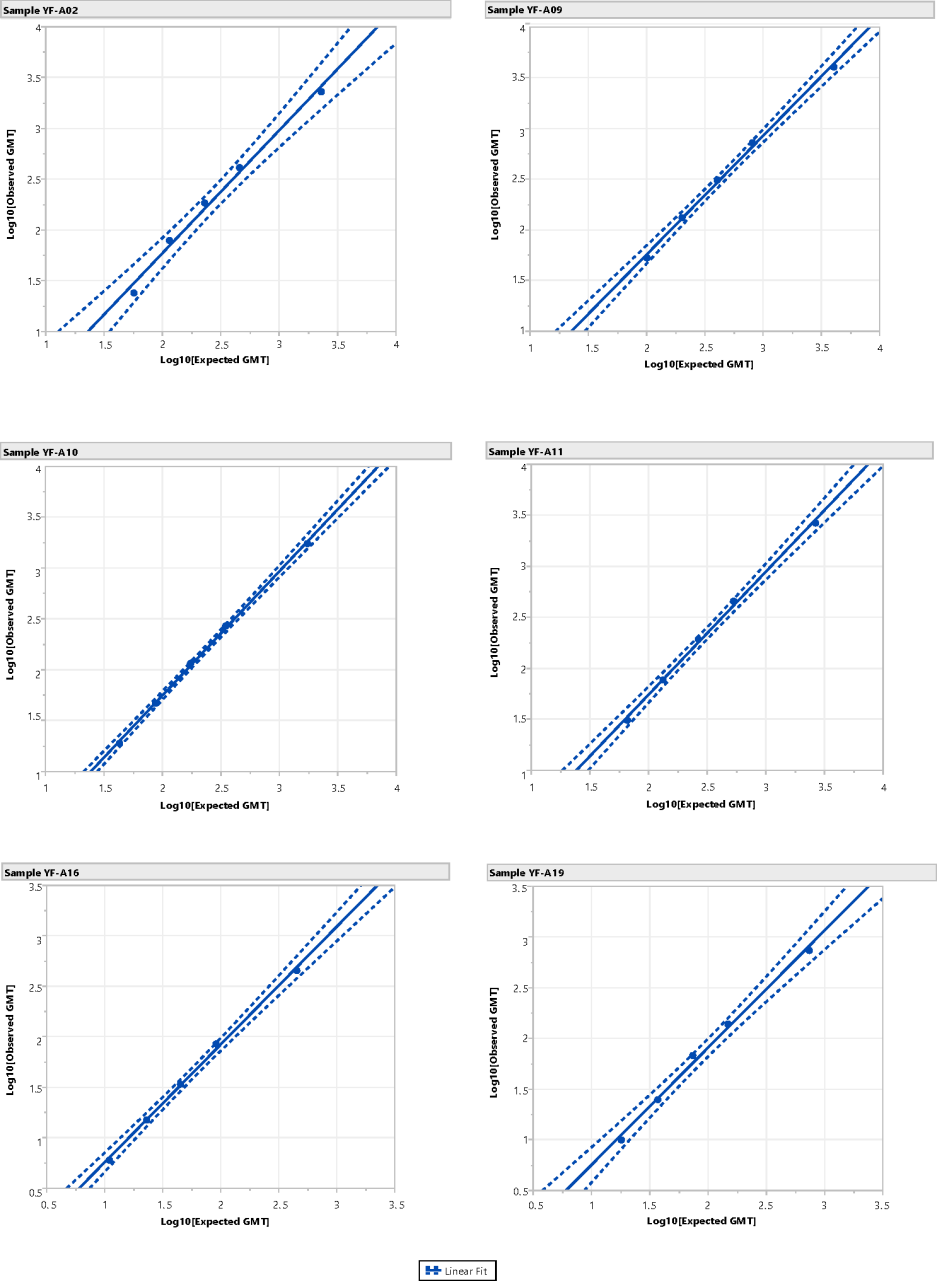
The YF MN assay linear regression analysis was performed to assess the dilutional linearity of the method. Each of the six human serum samples (undiluted and pre-diluted 1:5, 1:10, 1:20, and 1:40) was tested in triplicate in three individual assay runs. One hundred percent (6/6) of the samples had an R^2^ > 0.95 and a slope between 0.67 and 1.50. The y-axis represents the log-transformed observed GMTs. The x-axis represents the log-transformed expected GMTs. The blue shaded area represents the confidence interval of the “expected” y at the given x. Abbreviations: GMT, geometric mean titer; MN, microneutralization; and YF, yellow fever.

The YF MN assay demonstrated suitable linearity for the detection of YF virus-neutralizing antibodies; the R^2^ value for the log–linear regression analysis was >0.95, and the slope was within the range of 0.67–1.50 for 100% (6/6) of samples (Table 6; Fig. 2).

#### Specificity

All samples spiked with anti-DENV antibody, anti-Japanese encephalitis virus (JEV) antibody, and anti-Zika virus (ZIKV) antibody had an absolute difference between the observed and expected log_2_ titers of <1.00 (Table 6). Similarly, all samples spiked into hemolytic, lipemic, and icteric serum matrices had an absolute difference of the log_2_-observed and log_2_-expected titer of <1.00 (Table 6; Table S4). Thus, the YF MN assay demonstrated suitable specificity in the presence of other anti-flavivirus antibodies and across serum matrices.

## DISCUSSION

This study describes the validation of a new, cell-based YF MN assay for the detection and quantification of YF virus-neutralizing antibodies. We demonstrated that the YF MN assay is suitable for the detection and quantification of YF virus-neutralizing antibodies in human samples, meeting all the acceptance criteria for intra-assay precision (repeatability), intermediate precision, dilutional accuracy, linearity, and specificity, indicative of a robust assay.

To ensure the validity of the results, during the development and validation of the YF MN assay, valid titer ranges for both positive and negative IQCs were established, and the short-term stability of YF virus-neutralizing antibodies in human serum samples under various conditions was confirmed. Further, the YF MN assay demonstrated low cross-reactivity for anti-DENV antibodies, with very low titers observed. The lack of a link between DENV titers and cross-detection suggests a limited impact on the outcome of epidemiologic and clinical studies focusing on YF.

Although the PRNT is highly specific for the differentiation of orthoflavivirus infections, cross-reactivity among orthoflaviviruses remains an issue due to high levels of protein sequence homology (15, 20–22). The YF MN assay described here combines a high degree of assay specificity with an acceptable level of assay sensitivity for the detection and quantification of YF virus-neutralizing antibodies. For samples from those with a history of YF vaccination, 100% serostatus agreement was observed when comparing neutralizing antibody titers measured with the YF MN assay versus the YF PRNT_50_, demonstrating acceptable assay sensitivity. Further, a comparison of neutralizing antibody titers measured with the YF PRNT_80_, using a more stringent neutralization endpoint of 80%, versus the YF MN assay, resulted in 100% serostatus agreement in YF- naïve participants, demonstrating high assay specificity.

Additionally, it has been previously noted that storage conditions can impact YF virus-neutralizing antibody titers, highlighting the importance of detailing the collection and storage of samples in the methods sections of all YF virus studies (23). However, in this study, we demonstrated that multiple freeze/thaw cycles and storage at 4°C had a limited impact on the stability of YF-neutralizing antibodies.

Notably, the YF MN assay validation has been reviewed by the Center for Biologics Evaluation and Research (CBER) and is considered suitable for the detection and quantification of YF-neutralizing antibody titers in vaccine clinical trials. Thus, the validated cell-based YF MN assay is suitable for detecting and measuring YF virus-neutralizing antibodies.

## MATERIALS AND METHODS

### Serum samples

IQC human serum samples were obtained from three healthy adult donors. The donors for IQC1 and IQC2 were immunized with the licensed YF-VAX® vaccine (Sanofi Pasteur, Swiftwater, PA, USA) and were positive for YF virus-neutralizing antibodies. The donor for IQC3 was not immunized with a YF vaccine (YF-naïve) and was negative for YF virus-neutralizing antibodies.

To compare serostatus agreement between the YF MN assay and YF PRNT, serum samples were obtained from healthy donors (employees, Sanofi, Swiftwater, PA, USA) with and without a history of immunization with YF-VAX and from a previous phase II clinical study (NCT01488890) (24).

To assess the cross-reactivity of the method, anti-DENV antibody-positive human serum samples (*n* = 38) were obtained from naturally DENV-infected donors (Sanofi CYD47 clinical study [NCT01550289], SeraCare [Honduras], and Biomnis [San Salvador]).

To assess the specificity of the method, an anti-DENV antibody-positive human serum sample (Biomnis, Lyon, France), an anti-JEV antibody-positive human serum sample from a previously vaccinated donor, and an anti-ZIKV antibody-positive human serum sample (ABO Pharmaceuticals, San Diego, CA, USA) were evaluated.

Ig-depleted human sera were obtained commercially from BBI Solutions (Crumlin, UK).

Samples were obtained in compliance with Health Insurance Portability and Accountability regulations and Sanofi policies and procedures (13). For the samples provided by Sanofi employees, consent was obtained prior to collection.

### YF virus

Working lots of YF virus (YF-17D strain) were produced in serum-free Vero cells (Sanofi, Marcy L’Etoile, France or Neuville, France) and stored as single-use aliquots at –80°C to –40°C.

### YF PRNT assay procedure

Serial two-fold dilutions of heat-inactivated serum samples were incubated with a constant challenge dose of YF virus. Vero cells (ATCC® CCL-81™) were seeded into 24-well plates at a concentration of 1.5 × 10^5^ cells per well one day before use. The serum samples were tested at an initial 1:5 dilution, with an equal volume of YF virus, resulting in a final serum starting dilution of 1:10. All plates were incubated at 4°C for 18 h. The serum-virus mixture was then used to inoculate pre-seeded Vero cell monolayers, and following the addition of an overlay, the infected cells were incubated for five days at 37°C with 5% CO_2_. To visualize YF virus plaques, Vero cell monolayers were washed, fixed, and stained with a crystal violet solution. Plaques were counted to calculate the YF PRNT_50_ or YF PRNT_80_ neutralizing antibody titer, which was defined as the reciprocal of the highest test serum dilution for which virus infectivity was reduced by 50% or 80%, respectively, relative to the challenge virus dose. YF PRNT was performed by IQVIA Laboratories (Cypress, CA, USA) under contract by Sanofi.

### YF MN assay procedure

Serial two-fold dilutions of heat-inactivated serum samples were incubated with a challenge dose targeting 100 50% tissue culture infectious dose (TCID_50_) of YF virus. Vero cells (ATCC® CCL-81™) were seeded into 96-well microplates at a concentration of 2.0 × 10^4^ cells per well one day before use. The TCID_50_ plate included eight replicates of the YF virus challenge dose diluted two-fold, 11 times, horizontally across the 96-well plate. The sample plate(s) included single serum samples with 11 two-fold serial dilutions horizontally across a 96-well sample plate. Serum samples were tested at an initial 1:5 dilution with an equal volume of YF virus, resulting in a final serum starting dilution of 1:10. Each plate included four wells of cell controls (no virus) and four wells of virus controls (no serum). All plates were incubated at 37°C with 5% CO_2_ for 120 min. The serum–virus mixtures were inoculated into wells of 96-well plates with preformed Vero cell monolayers and adsorbed at 37°C with 5% CO_2_ for 60 min. Additional assay media was added to all wells without removing the existing inoculum and incubated at 37°C with 5% CO_2_ for 2 days. After washing and fixation of the Vero cell monolayers, YF antigen production in cells was detected by successive incubations with an anti-flavivirus envelope-protein mouse monoclonal antibody (HB112-4G2; Biotem, Apprieu, France), horseradish peroxidase IgG conjugate (Jackson ImmunoResearch Laboratories, West Grove, PA, USA), and a chromogenic substrate. The resulting optical density (OD; 450 nm) was measured using a Molecular Devices SPECTRAmax Plus 384 plate reader (Molecular Devices, LLC, San Jose, CA, USA).

### Calculation of YF virus neutralizing antibody titers

The 50% neutralizing titer (MN_50_) of the test serum sample against the YF virus was defined as the reciprocal of the test serum dilution for which virus infectivity was reduced by 50% (50% neutralizing point) relative to the challenge YF virus control (without serum). The 50% neutralization point for each test plate was calculated using:


[(Average OD of virus control − Average OD of cell control)/2 + Average OD of cell control].

The YF virus neutralizing antibody titer for each sample was interpolated by calculating the slope and intercept using the last dilution with an OD below the 50% neutralization point and the first dilution with an OD above the 50% neutralization point. The MN_50_ titer was determined using the following calculation:


(OD of 50% neutralization point − intercept)/slope.

### Qualification of IQCs

Qualification of IQCs was performed to determine the suitability of each sample serum to be used to determine the validity of an assay run. Candidate human serum samples were tested as unknown samples, generating a minimum of 30 titers. For IQCs positive for YF virus-neutralizing antibodies, ≥90% of the results were required to be within an acceptable titer range, based on assay variability (±2-fold from the GMT). For IQCs negative for YF virus-neutralizing antibodies, the target titer was required to be <10, with ≥90% of the results below the established LLOQ of the assay (<10). The Shapiro–Wilks test and normal quantile–quantile plot were used to check normal distribution.

### Short-term stability of YF virus-neutralizing antibodies

Sets of aliquots of test serum samples, including IQC samples, were prepared and assessed under various stability conditions, including freeze–thaw (five cycles), and stored at 2°C to 8°C for up to 14 days. The samples were tested using the YF MN assay alongside a set of samples that had been thawed just prior to testing (no treatment). Results for treated samples were compared with the results obtained without treatment.

### Cross-reactivity assessment

To evaluate the cross-reactivity of the YF MN assay for DENV antibodies, 38 samples from individuals with naturally occurring DENV infection from India (*n* = 35), San Salvador (*n* = 2), and Honduras (*n* = 1) were tested using both the YF MN assay and a previously validated DENV PRNT (25).

### YF MN assay validation

A summary of YF MN assay validation parameters and acceptance criteria can be found in Table 1. Briefly, the following parameters were assessed: intra-assay precision (repeatability), intermediate precision, dilutional accuracy, linearity, specificity, confirmation of the upper limit of quantification (ULOQ), and confirmation of LLOQ. For intra-assay precision (repeatability) and intermediate precision, the observed titers were considered dependent variables, and the sample and assay runs were considered independent variables. Repeatability and intermediate precision were evaluated by calculating the variance component using a mixed model as follows:


$$y_{ijk}=\mu +\alpha_{i}+run_{j\left(i\right)}+rep_{k\left(j\left(i\right)\right)}+\varepsilon_{ijk},$$

where *y_ijk_* was the observed result in the log-scale; *α_i_* $\left(\sum_{i}\alpha_{i}=0\right)$ was the constant difference between the mean of the *i*th sample and mean of the panel (*μ*); and *run* and *rep* represented the runs of the YF MN assay, which were replicated within each run. Additionally, *α_I_* was a fixed effect in the mixed model, and *run* and *rep* were both random effects following normal distributions:


$$run_{j(i)}∼N(0,\sigma_{run}^{2}),rep_{k(j(i))}∼N(0,\sigma_{rep}^{2}),\varepsilon_{ijk}p_{k(j(i))}∼∼N(0,\sigma^{2}).$$

The variance component of repeatability and intermediate precision were estimated as follows:


$${\overset{}{\hat{\sigma }}}_{rep}^{2}+{\overset{}{\hat{\sigma }}}_{run}^{2}\;and\;{\overset{}{\hat{\sigma }}}_{rep}^{2}+{\overset{}{\hat{\sigma }}}_{run}^{2}+\overset{}{\hat{\sigma }},$$

where ${\hat{\sigma }}_{rep}^{2}$, ${\hat{\sigma }}_{run}^{2}$, and ${\hat{\sigma }}^{2}$ were variance component estimators of *σ^2^_rep_*, *σ^2^_run_*, and *σ^2^*, respectively. Therefore, the intra-assay precision and intermediate precision % GCV were calculated as


$$100\%\times \left(\sqrt{e^{{\overset{}{\hat{\sigma }}}_{rep}^{2}+{\overset{}{\sigma }}^{2}}-1}\right)\;and\;100\%\times \left(\sqrt{e^{{\overset{}{\hat{\sigma }}}_{rep}^{2}+{\overset{}{\hat{\sigma }}}_{run}^{2}+{\overset{}{\hat{\sigma }}}^{2}}-1}\right),\;respectively.$$

The ULOQ was defined by testing at a serum starting dilution of 1:20 (twice the method starting dilution), and the LLOQ was defined by testing at a serum starting dilution of 1:5 (half the method starting dilution). Dilutional accuracy and linearity were tested using undiluted and pre-diluted (1:5, 1:10, 1:20, and 1:40) serum samples.

To assess specificity, two different approaches were employed: spiking and matrix effect assessment. For specificity spiking, YF virus-positive human samples were spiked with anti-DENV, anti-JEV, and anti-ZIKV antibodies. For the matrix effect assessment, YF virus-positive human samples were spiked into different matrix samples. The matrix samples were generated by taking anti-YF virus negative human serum and spiking with approximately 0.5 mg/mL of hemolysate for the hemolytic samples (Sun Diagnostics, New Gloucester, ME, USA), approximately 0.75 mg/mL of triglyceride-rich lipoproteins for the lipemic samples (Sun Diagnostics, New Gloucester, ME, USA), and approximately 0.02 mg/mL of conjugated bilirubin for the icteric samples (Sun Diagnostics, New Gloucester, ME, USA).

## SUPPLEMENTAL MATERIAL

Supplemental material spectrum.03348-24-s0001.docx Fig. S1; Tables S1 to S4.

ASM does not own the copyrights to Supplemental Material that may be linked to, or accessed through, an article. The authors have granted ASM a non-exclusive, world-wide license to publish the Supplemental Material files. Please contact the corresponding author directly for reuse.
