# Supplementary material for: The development and validation of a microneutralization assay for the detection and quantification of anti-yellow fever virus antibodies in human serum
Source: Microbiol Spectr. 2025 Mar 4;13(4):e03348-24. doi: 10.1128/spectrum.03348-24 (PMC11960057; doi:10.1128/spectrum.03348-24)
Supplement: Supplemental material — Fig. S1; Tables S1 to S4. [file spectrum.03348-24-s0001.docx]

**Supplemental material**


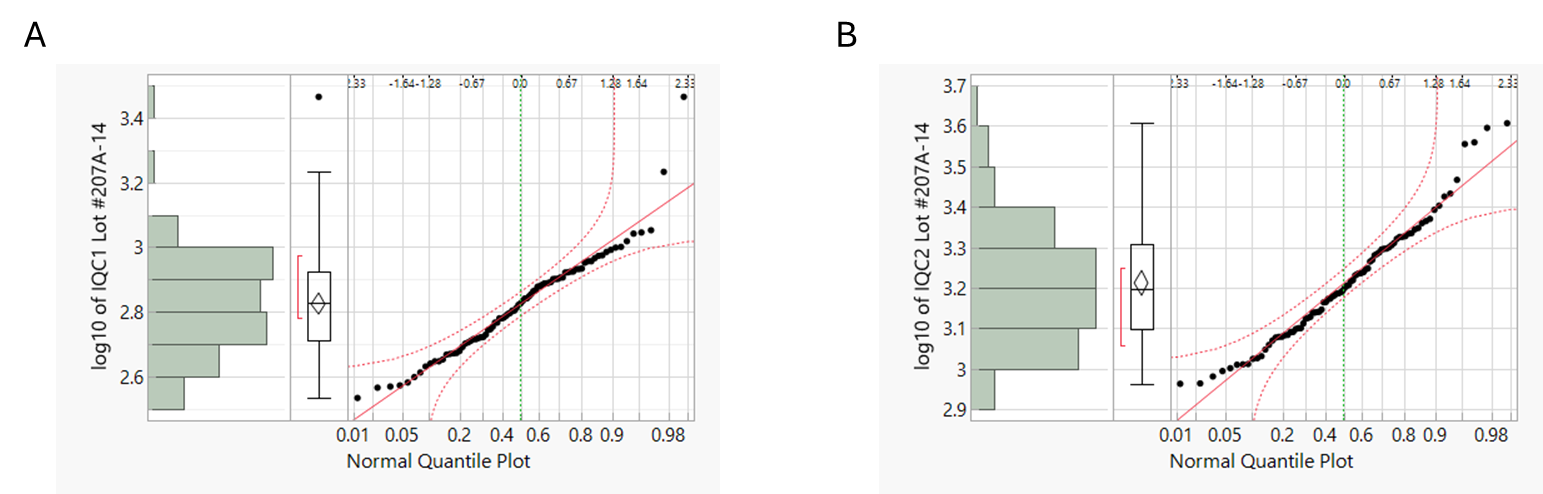


Fig S1 Histogram and normality assessment of IQC1 (A) and ICQ2 (B). The y-axis on the histograms is the observed YF virus MN_50_ titers.

The x-axis on the quantile–quantile plots is the quantile from 0 to 1. Outliers are not shown in the histogram but are shown in the quantile–quantile plot. Abbreviations: IQC, internal quality control; MN_50_, 50% microneutralization; YF, yellow fever.

**Table S1. YF MN assay, PRNT_50_, and PRNT_80_ results from human serum samples from participants with a history of YF vaccination**

| **Sample ID** | **YF MN GMT (n=3)** | **YF PRNT_50_ GMT (n=1–3)** | **YF PRNT_80_ GMT (n=1–3)** |
| --- | --- | --- | --- |
| YFQ-801^a^ | 396 | 160 | 50 |
| YFQ-802^a^ | 408 | 202 | 101 |
| YFQ-803^a^ | 1,327 | 127 | 40 |
| YFQ-805^a^ | 309 | 50 | 20 |
| YFQ-806^a^ | 301 | 160 | 57 |
| YFQ-807^a^ | 174 | 160 | 50 |
| YFQ-808^a^ | 84 | 95 | 28 |
| YFQ-809^a^ | 40 | 80 | 24 |
| YFQ-810^a^ | 189 | 57 | 20 |
| YFQ-813^a^ | 440 | 202 | 80 |
| YFQ-814^a^ | 715 | 127 | 32 |
| YFQ-815^a^ | 940 | 508 | 160 |
| YFQ-816^a^ | 795 | 381 | 113 |
| YFQ-817^a^ | 431 | 202 | 80 |
| YFQ-818^a^ | 764 | 320 | 160 |
| YFQ-819^a^ | 40 | 80 | 20 |
| YFQ-820^a^ | 1,534 | 640 | 320 |
| YFQ-821^a^ | 1,524 | 508 | 202 |
| YFQ-822^a^ | 406 | 40 | 16 |
| YFQ-823^a^ | 745 | 320 | 80 |
| YFQ-824^a^ | 189 | 101 | 13 |
| YFQ-825^a^ | 1,858 | 3,620 | 320 |
| YFQ-826^a^ | 830 | 80 | 14 |
| YFQ-827^a^ | 25 | 32 | <10 |
| YFQ-828^a^ | 108 | 80 | 16 |
| YFQ-829^a^ | 1,253 | 538 | 113 |
| YFQ-830^a^ | 961 | 640 | 190 |
| YFQ-831^a^ | 28 | 50 | 13 |
| YFQ-832^a^ | 129 | 113 | 28 |
| YFQ-833^a^ | 687 | 320 | 80 |
| YFQ-834^a^ | 248 | 160 | 67 |
| YFQ-836^a^ | 754 | 127 | 40 |
| YFQ-837^a^ | 93 | 63 | 25 |
| YFQ-838^a^ | 23 | 32 | <10 |
| YFQ-840^a^ | 745 | 538 | 160 |
| YF MN 1^b^ | 2,357 | 10,240 | 1,280 |
| YF MN 2^b^ | 2,769 | 10,240 | 320 |
| YF MN 3^b^ | 562 | 320 | 40 |
| YF MN 4^b^ | 955 | 2,560 | 320 |
| YF MN 5^b^ | 935 | 1,280 | 320 |
| YF MN 6^b^ | 2,026 | 5,120 | 1,280 |
| YF MN 7^b^ | 707 | 320 | 160 |
| YF MN 8^b^ | 2,709 | 10,240 | 1,280 |
| YF MN 9^b^ | 480 | 320 | 80 |
| YF MN 11^b^ | 3,515 | 5,120 | 320 |
| YF MN 13^b^ | 140 | 160 | 40 |
| YF MN 14^b^ | 242 | 320 | 40 |
| YF MN 15^b^ | 257 | 5,120 | 160 |
| YF MN 16^b^ | 489 | 5,120 | 320 |
| YF MN 17^b^ | 1,190 | 5,120 | 640 |
| YF MN 18^b^ | 440 | 320 | 160 |
| YF MN 19^b^ | 140 | 160 | 40 |
| YF MN 20^b^ | 20 | 40 | <10 |
| YF MN 21^b^ | 278 | 1,280 | 160 |
| YF MN 24^b^ | 207 | 320 | 80 |
| YF MN 25^b^ | 342 | 320 | 320 |
| YF MN 26^b^ | 756 | 2,560 | 320 |
| YF MN 27^b^ | 1,594 | 640 | 80 |
| YF MN 29^b^ | 2,846 | 5,120 | 1,280 |
| YF MN 30^b^ | 1,295 | 10,240 | 1,280 |
| YF MN 31^b^ | 2,409 | 10,240 | 1,280 |
| YF MN 32^b^ | 1,863 | 1,280 | 160 |
| YF MN 33^b^ | 2,403 | 5,120 | 80 |
| YF MN 34^b^ | 902 | 2,560 | 160 |
| YF MN 36^b^ | 204 | 160 | 20 |
| YF MN 37^b^ | 363 | 320 | 40 |
| YF MN 38^b^ | 294 | 160 | 40 |
| YF MN 41^b^ | 195 | 320 | 40 |
| YF MN 42^b^ | 590 | 640 | 160 |
| YF MN 43^b^ | 133 | 160 | 40 |
| YF MN 44^b^ | 1,630 | 2,560 | 160 |
| YF MN 45^b^ | 767 | 1,280 | 320 |
| YF MN 46^b^ | 854 | 2,560 | 160 |
| YF MN 47^b^ | 1,687 | 5,120 | 1,280 |
| YF MN 48^b^ | 671 | 2,560 | 320 |
| YF MN 49^b^ | 484 | 640 | 160 |
| YF MN 50^b^ | 1,283 | 5,120 | 1,280 |
| YF MN 51^b^ | 352 | 320 | 80 |
| YF MN 52^b^ | 188 | 160 | 10 |
| YF MN 54^b^ | 6,551 | >20,480 | 1,280 |
| YF MN 55^b^ | 1,246 | 10,240 | 320 |
| YF MN 56^b^ | 144 | 160 | 40 |
| YF MN 57^b^ | 278 | 80 | 40 |
| YF MN 59^b^ | 537 | 640 | 160 |
| YF MN 61^b^ | 253 | 160 | 80 |
| YF MN 62^b^ | 7,734 | >20,480 | 2,560 |
| YF MN 63^b^ | 281 | 2,560 | 160 |
| YF MN 65^b^ | 1,531 | 2,560 | 160 |
| YF MN 68^b^ | 247 | 160 | 20 |
| YF MN 69^b^ | 1,969 | 10,240 | 640 |
| YF MN 70^b^ | 889 | 1,280 | 160 |
| YF MN 73^b^ | 665 | 1,280 | 80 |
| YF MN 74^b^ | 1,331 | 2,560 | 640 |
| YF MN 75^b^ | 580 | 1,280 | 320 |
| YF MN 76^b^ | 27 | 40 | <10 |
| YF MN 77^b^ | 476 | 1,280 | 80 |
| YF MN 78^b^ | 905 | 5,120 | 320 |
| YF MN 79^b^ | 1,451 | 2,560 | 320 |
| YF MN 80^b^ | 273 | 80 | <10 |
| YF MN 81^b^ | 160 | 160 | 40 |
| YF MN 82^b^ | 695 | 320 | 80 |
| YF MN 83^b^ | 141 | 160 | 20 |
| YF MN 84^b^ | 538 | 640 | 160 |
| YF MN 86^b^ | 2,052 | 2,560 | 320 |
| YF MN 87^b^ | 1,308 | 1,280 | 80 |
| YF MN 88^b^ | 946 | 5,120 | 160 |
| YF MN 89^b^ | 626 | 1,280 | 320 |
| YF MN 90^b^ | 750 | 1,280 | 320 |
| YF MN 91^b^ | 280 | 320 | 80 |
| YF MN 92^b^ | 470 | 160 | 80 |
| YF MN 94^b^ | 4,262 | 1,280 | 160 |
| YF MN 95^b^ | 1,937 | 5,120 | 640 |
| YF MN 96^b^ | 307 | 320 | 160 |
| YF MN 97^b^ | 918 | 5,120 | 160 |
| YF MN 98^b^ | 662 | 640 | 160 |
| YF MN 100^b^ | 102 | 80 | 20 |
| YF MN 101^b^ | 5,778 | 5,120 | 160 |
| YF MN 102^b^ | 794 | 5,120 | 160 |
| YF MN 104^b^ | 3,150 | 5,120 | 640 |
| YF MN 106^b^ | 400 | 1,280 | 80 |
| YF MN 107^b^ | 360 | 160 | 80 |
| YF MN 108^b^ | 838 | 2,560 | 160 |
| YF MN 111^b^ | 68 | 80 | 20 |
| YF MN 112^b^ | 400 | 2,560 | 320 |
| YF MN 113^b^ | 1,340 | 2,560 | 160 |
| YF MN 114^b^ | 800 | 1,280 | 160 |
| YF MN 116^b^ | 117 | 160 | 40 |
| YF MN 117^b^ | 942 | 2,560 | 160 |
| YF MN 119^b^ | 86 | 160 | 40 |
| YF MN 121^b^ | 262 | 1,280 | 160 |
| YF MN 122^b^ | 788 | 2,560 | 320 |
| YF MN 123^b^ | 728 | 1,280 | 160 |
| YF MN 124^b^ | 355 | 160 | 80 |
| YF MN 125^b^ | 1,072 | 5,120 | 320 |
| YF MN 126^b^ | 795 | 640 | 320 |
| YF MN 128^b^ | 748 | 2,560 | 640 |
| YF MN 129^b^ | 256 | 320 | 160 |
| YF MN 130^b^ | 657 | 2,560 | 640 |
| YF MN 131 ^b^ | 796 | 5,120 | 160 |
| YF MN 132^b^ | 210 | 320 | 160 |
| YF MN 133^b^ | 101 | 160 | 40 |
| YF MN 137^b^ | 973 | 5,120 | 320 |

^a^Sanofi employee healthy adult donors

^b^Sanofi CYD51 clinical study participants

Abbreviations: GMT, geometric mean titer; MN, microneutralization; PRNT_50_, 50% plaque reduction neutralization test; PRNT_80_, 80% plaque reduction neutralization test; YF, yellow fever.

**Table S2. YF MN assay, PRNT_50_, and PRNT_80_ results from human serum samples from participants without a history of YF vaccination**

| **Sample ID** | **YF MN GMT (n=3)** | **YF PRNT_50_ GMT (n=1–3)** | **YF PRNT_80_ titer GMT (n=1–3)** |
| --- | --- | --- | --- |
| YF MN 10^a^ | <10 | <10 | <10 |
| YF MN 12^a^ | <10 | <10 | <10 |
| YF MN 22^a^ | <10 | <10 | <10 |
| YF MN 23^a^ | <10 | <10 | <10 |
| YF MN 28^a^ | <10 | <10 | <10 |
| YF MN 35^a^ | <10 | <10 | <10 |
| YF MN 39^a^ | <10 | <10 | <10 |
| YF MN 40^a^ | <10 | <10 | <10 |
| YF MN 53^a^ | <10 | <10 | <10 |
| YF MN 58^a^ | <10 | <10 | <10 |
| YF MN 60^a^ | <10 | <10 | <10 |
| YF MN 64^a^ | <10 | <10 | <10 |
| YF MN 66^a^ | <10 | <10 | <10 |
| YF MN 67^a^ | <10 | <10 | <10 |
| YF MN 71^a^ | <10 | <10 | <10 |
| YF MN 72^a^ | <10 | <10 | <10 |
| YF MN 85^a^ | <10 | <10 | <10 |
| YF MN 93^a^ | <10 | <10 | <10 |
| YF MN 99^a^ | <10 | <10 | <10 |
| YF MN 103^a^ | <10 | <10 | <10 |
| YF MN 105^a^ | <10 | <10 | <10 |
| YF MN 109^a^ | <10 | <10 | <10 |
| YF MN 110^a^ | <10 | <10 | <10 |
| YF MN 115^a^ | <10 | <10 | <10 |
| YF MN 118^a^ | <10 | <10 | <10 |
| YF MN 120^a^ | <10 | <10 | <10 |
| YF MN 127^a^ | <10 | <10 | <10 |
| YF MN 134^a^ | <10 | <10 | <10 |
| YF MN 135^a^ | <10 | <10 | <10 |
| YF MN 136^a^ | <10 | <10 | <10 |
| YF MN 200^a^ | <10 | 20 | <10 |
| YF MN 201^a^ | <10 | 20 | <10 |
| YF MN 202^a^ | <10 | 20 | <10 |
| YF MN 203^a^ | <10 | 160 | <10 |
| YF MN 204^a^ | <10 | 40 | <10 |
| YF MN 205^a^ | <10 | 20 | <10 |
| YF MN 206^a^ | <10 | 10 | <10 |
| YF MN 207^a^ | <10 | 40 | <10 |
| YF MN 208^a^ | <10 | 80 | <10 |
| YF MN 209^a^ | <10 | 40 | <10 |
| YF MN 210^a^ | <10 | 20 | <10 |
| YF MN 211^a^ | <10 | 40 | <10 |
| YF MN 212^a^ | <10 | 20 | <10 |
| YF MN 213^a^ | <10 | 80 | <10 |
| YF MN 214^a^ | <10 | 80 | <10 |
| YF MN 215^a^ | <10 | 20 | <10 |
| YF MN 216^a^ | <10 | 40 | <10 |
| YF MN 217^a^ | <10 | 20 | <10 |
| YF MN 218^a^ | <10 | 80 | <10 |
| YF MN 219^a^ | <10 | 80 | <10 |
| YF MN 220^a^ | <10 | 80 | <10 |
| YF MN 221^a^ | <10 | 40 | <10 |
| YF MN 222^a^ | <10 | 20 | <10 |
| YF MN 223^a^ | <10 | 40 | <10 |
| YF MN 224^a^ | <10 | 160 | <10 |
| YF MN 225^a^ | <10 | 10 | <10 |
| YF MN 226^a^ | <10 | 20 | <10 |
| YF MN 227^a^ | <10 | 80 | <10 |
| YF MN 228^a^ | <10 | 20 | <10 |
| YF MN 229^a^ | <10 | 80 | <10 |
| YF MN 230^a^ | <10 | 20 | <10 |
| YF MN 231^a^ | <10 | 20 | <10 |
| YF MN 232^a^ | <10 | 20 | <10 |
| YF MN 233^a^ | <10 | 20 | <10 |
| YF MN 234^a^ | <10 | 40 | <10 |
| YF MN 235^a^ | <10 | 20 | <10 |
| YF MN 236^a^ | <10 | 20 | <10 |
| YF MN 237^a^ | <10 | 20 | <10 |
| YF MN 238^a^ | <10 | 20 | <10 |
| YF MN 239^a^ | <10 | 40 | <10 |
| YF MN 240^a^ | <10 | 20 | <10 |
| YF MN 241^a^ | <10 | 20 | <10 |
| YF MN 242^a^ | <10 | 40 | <10 |
| YF MN 243^a^ | <10 | 40 | <10 |
| YF MN 244^a^ | <10 | 80 | <10 |
| YF MN 245^a^ | <10 | 20 | <10 |
| YF MN 246^a^ | <10 | 20 | <10 |
| YF MN 247^a^ | <10 | 80 | <10 |
| YF MN 248^a^ | <10 | 20 | <10 |
| YF MN 249^a^ | <10 | 20 | <10 |
| YF MN 250^a^ | <10 | 40 | <10 |
| YF MN 251^a^ | <10 | 20 | <10 |
| YF MN 252^a^ | <10 | 40 | <10 |
| YF MN 253^a^ | <10 | 20 | <10 |
| YF MN 254^a^ | <10 | 20 | <10 |
| YF MN 255^a^ | <10 | 20 | <10 |
| YF MN 256^a^ | <10 | 10 | <10 |
| YF MN 257^a^ | <10 | 80 | <10 |
| YF MN 258^a^ | <10 | 20 | <10 |
| YF MN 259^a^ | <10 | 20 | <10 |
| YF MN 260^a^ | <10 | 40 | <10 |
| YF MN 261^a^ | <10 | 20 | <10 |
| YFQ-804^b^ | <10 | <10 | <10 |
| YFQ-835^b^ | <10 | <10 | <10 |

^a^Sanofi CYD51 clinical study participants

^b^Sanofi employee healthy adult donors

Abbreviations: GMT, geometric mean titer; MN, microneutralization; PRNT_50_, 50% plaque reduction neutralization test; PRNT_80_, 80% plaque reduction neutralization test; YF, yellow fever.

**Table S3. Anti-YF virus MN antibodies and anti-DENV PRNT antibodies in naturally DENV-infected human samples**

| **Sample #** | **Anti-DENV status** | **Anti-YF status** | **Anti-YF virus MN titer** | **Anti-DENV PRNT_50_ titers** | | | |
| --- | --- | --- | --- | --- | --- | --- | --- |
|  |  |  |  | **DENV-1** | **DENV-2** | **DENV-3** | **DENV-4** |
| 1^a^ | Positive | Assumed negative | 13 | 261 | 1,354 | 261 | 280 |
| 2^a^ | Positive | Assumed negative | <10 | 3,338 | 2,334 | 1,192 | 440 |
| 3^a^ | Positive | Assumed negative | <10 | 7,863 | 3,301 | 2,391 | 942 |
| 4^a^ | Positive | Assumed negative | <10 | 4,736 | 697 | 2,335 | 78 |
| 5^a^ | Positive | Assumed negative | <10 | 2,922 | 427 | 1,242 | 186 |
| 6^a^ | Positive | Assumed negative | <10 | 1,955 | 957 | 472 | 250 |
| 7^a^ | Positive | Assumed negative | <10 | 1,656 | 792 | 421 | 156 |
| 8^a^ | Positive | Assumed negative | <10 | 156 | 132 | 430 | 146 |
| 9^a^ | Positive | Assumed negative | <10 | 2,486 | 865 | 920 | 260 |
| 10^a^ | Positive | Assumed negative | <10 | 189 | 96 | 144 | 18 |
| 11^a^ | Positive | Assumed negative | 15 | 259 | 812 | 283 | 205 |
| 12^a^ | Positive | Assumed negative | <10 | 6,740 | 1,152 | 1,627 | 97 |
| 13^a^ | Positive | Assumed negative | <10 | 1,309 | 4,146 | 4,580 | 116 |
| 14^a^ | Positive | Assumed negative | <10 | 48 | 419 | 70 | 36 |
| 15^a^ | Positive | Assumed negative | <10 | 88 | 76 | 92 | 348 |
| 16^a^ | Positive | Assumed negative | <10 | 71 | 92 | 664 | <10 |
| 17^a^ | Positive | Assumed negative | <10 | 307 | 1,228 | 2,321 | 122 |
| 18^a^ | Positive | Assumed negative | <10 | 296 | 331 | 6,189 | 121 |
| 19^a^ | Positive | Assumed negative | 15 | 619 | 1,213 | 378 | 270 |
| 20^a^ | Positive | Assumed negative | 11 | 594 | 2,229 | 384 | 205 |
| 21^a^ | Positive | Assumed negative | <10 | 152 | 1,488 | 136 | 93 |
| 22^a^ | Positive | Assumed negative | <10 | 98 | 800 | 363 | 57 |
| 23^a^ | Positive | Assumed negative | <10 | 102 | 290 | 371 | 43 |
| 24^a^ | Positive | Assumed negative | <10 | 355 | 326 | 2,414 | 103 |
| 25^a^ | Positive | Assumed negative | <10 | 888 | 169 | 363 | 37 |
| 26^a^ | Positive | Assumed negative | 17 | 716 | 815 | 416 | 445 |
| 27^a^ | Positive | Assumed negative | <10 | 186 | 815 | 39 | 28 |
| 28^a^ | Positive | Assumed negative | <10 | 235 | 3,688 | 775 | 150 |
| 29^a^ | Positive | Assumed negative | <10 | 2,683 | 132 | 361 | 145 |
| 30^a^ | Positive | Assumed negative | <10 | 367 | 2,502 | 177 | 108 |
| 31^a^ | Positive | Assumed negative | <10 | 1,617 | 237 | 338 | 91 |
| 32^a^ | Positive | Assumed negative | <10 | 111 | 212 | 93 | 396 |
| 33^a^ | Positive | Assumed negative | <10 | 231 | 65 | 54 | 32 |
| 34^a^ | Positive | Assumed negative | <10 | 224 | 651 | 243 | 37 |
| 35^a^ | Positive | Assumed negative | <10 | 37 | 21 | 617 | <10 |
| 36^b^ | Positive | Assumed negative | <10 | 2,428 | 6,990 | 2,567 | 10,724 |
| 37^b^ | Positive | Assumed negative | <10 | 6,424 | 13,243 | 38,285 | 811 |
| 38^c^ | Positive | Assumed negative | <10 | 591 | 3,323 | 3,702 | 1,679 |

^a^Sanofi clinical study sample from India.

^b^Biomnis commercial sample from San Salvador.

^c^SeraCare commercial sample from Honduras.

Abbreviations: DENV, dengue virus; MN_50_, 50% microneutralization; PRNT_50_, 50% plaque reduction neutralization test; YF, yellow fever.

**Table S4. Anti-YF MN antibodies after specificity spiking and matrix effect**

| **Sample #** | **Sample ID** | **Titer 1** | **Titer 2** | **Titer 3** | **Observed GMT** | **Expected GMT** | **ABS *log_2_* Difference** |
| --- | --- | --- | --- | --- | --- | --- | --- |
| 1 | YF-S3-Neg | 1,149 | 1,292 | 574 | 948 | 948 | N/A |
|  | YF-S3-DENV | 1,172 | 1,014 | 1,061 | 1,080 |  | 0.19 |
|  | YF-S3-JEV | 864 | 766 | 1,012 | 875 |  | 0.12 |
|  | YF-S3-Zika | 1,091 | 633 | 1,144 | 924 |  | 0.04 |
|  | YF-S3-Hem | 774 | 842 | 1,136 | 905 |  | 0.07 |
|  | YF-S3-Lip | 700 | 868 | 1,207 | 902 |  | 0.07 |
|  | YF-S3-Ict | 864 | 1,027 | 1,182 | 1,016 |  | 0.10 |
| 2 | YF-S4-Neg | 396 | 529 | 405 | 439 | 439 | N/A |
|  | YF-S4-DENV | 602 | 675 | 392 | 542 |  | 0.30 |
|  | YF-S4-JEV | 612 | 548 | 280 | 455 |  | 0.05 |
|  | YF-S4-Zika | 324 | 317 | 432 | 354 |  | 0.31 |
|  | YF-S4-Hem | 439 | 262 | 471 | 378 |  | 0.22 |
|  | YF-S4-Lip | 296 | 315 | 399 | 334 |  | 0.40 |
|  | YF-S4-Ict | 471 | 469 | 584 | 505 |  | 0.20 |
| 3 | YF-S6-Neg | 351 | 502 | 684 | 494 | 494 | N/A |
|  | YF-S6-DENV | 572 | 659 | 1,234 | 775 |  | 0.65 |
|  | YF-S6-JEV | 732 | 548 | 425 | 554 |  | 0.17 |
|  | YF-S6-Zika | 601 | 475 | 297 | 439 |  | 0.17 |
|  | YF-S6-Hem | 609 | 363 | 301 | 405 |  | 0.29 |
|  | YF-S6-Lip | 742 | 353 | 397 | 470 |  | 0.07 |
|  | YF-S6-Ict | 434 | 370 | 498 | 431 |  | 0.20 |
| 4 | YF-S8-Neg | 196 | 269 | 275 | 244 | 244 | N/A |
|  | YF-S8-DENV | 386 | 585 | 462 | 471 |  | 0.95 |
|  | YF-S8-JEV | 266 | 279 | 291 | 278 |  | 0.19 |
|  | YF-S8-Zika | 294 | 256 | 237 | 261 |  | 0.10 |
|  | YF-S8-Hem | 273 | 245 | 334 | 282 |  | 0.21 |
|  | YF-S8-Lip | 264 | 215 | 453 | 295 |  | 0.28 |
|  | YF-S8-Ict | 300 | 281 | 495 | 347 |  | 0.51 |
| 5 | YF-S10-Neg | 929 | 1,019 | 1,534 | 1,132 | 1,132 | N/A |
|  | YF-S10-DENV | 1,074 | 1,618 | 1,835 | 1,472 |  | 0.38 |
|  | YF-S10-JEV | 1,173 | 1,220 | 1,590 | 1,315 |  | 0.22 |
|  | YF-S10-Zika | 1,359 | 1,201 | 1,694 | 1,404 |  | 0.31 |
|  | YF-S10-Hem | 1,756 | 1,259 | 882 | 1,249 |  | 0.14 |
|  | YF-S10-Lip | 1,712 | 1,397 | 788 | 1,235 |  | 0.13 |
|  | YF-S10-Ict | 1,784 | 837 | 1,116 | 1,186 |  | 0.07 |
| 6 | YF-S11-Neg | 3,170 | 1,857 | 2,891 | 2,572 | 2,572 | N/A |
|  | YF-S11-DENV | 2,235 | 2,143 | 4,364 | 2,755 |  | 0.10 |
|  | YF-S11-JEV | 1,588 | 2,177 | 4,411 | 2,480 |  | 0.05 |
|  | YF-S11-Zika | 1,876 | 2,421 | 4,653 | 2,765 |  | 0.10 |
|  | YF-S11-Hem | 3,774 | 2,292 | 3,320 | 3,062 |  | 0.25 |
|  | YF-S11-Lip | 3,686 | 2,247 | 2,117 | 2,598 |  | 0.01 |
|  | YF-S11-Ict | 3,354 | 1,903 | 2,577 | 2,543 |  | 0.02 |

ABS, absolute value; DENV, dengue virus; GMT, geometric mean titer; Hem, hemolytic; Ict, icteric; JEV, Japanese encephalitis virus; Lip, lipemic; MN, microneutralization; Neg, negative; S, sample; YF, yellow fever; Zika, ZIKA virus.
